# Plasticity of sex-biased aggression in response to the sex of territory intruders in an African cichlid fish, *Julidochromis marlieri* .

**DOI:** 10.17912/micropub.biology.001795

**Published:** 2025-10-28

**Authors:** Ry Dennis, Kelsey J Wood, Suzy CP Renn, Andrew P Anderson

**Affiliations:** 1 Biology, Reed College

## Abstract

Conspecific intruders represent unique threats to each sex in an established mating pair, where each member may respond differently to the intruder. We investigated how individuals from established pairs of *Julidochromis marlieri* , a biparental cichlid that forms female-larger pairs , respond to conspecific intruders of either sex. When males were the larger individual in the pair, they have increased responses to male intruders compared to female intruders; whereas when females were the larger individual, they did not change their behavior. We suggest the pressures on both sexes are different based on the natural history of the species.

**Figure 1. Response to Intruders by Sex:**
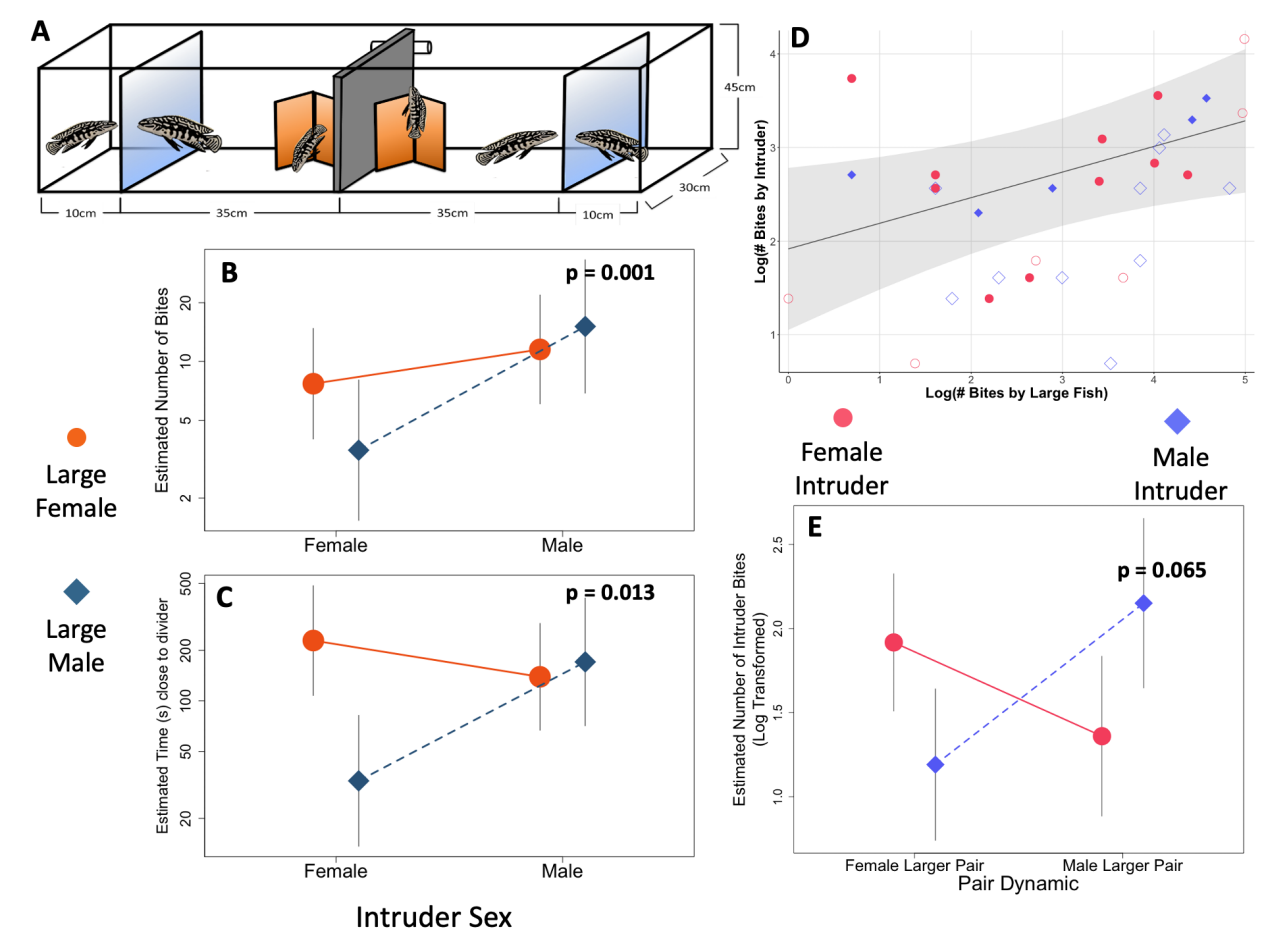
A) Observation tank setup pairs in compartments separated by an inserted opaque divider with an intruder in a compartment separated from a pair by perforated clear dividers. Reaction norms for individuals in response to the sex of the intruder. B) Number of Bites focal individual had toward the intruder. C) Time focal individual spent close to the divider separating it from the intruder. These values represent estimates from the model after all fixed and random effects were considered. Displayed p-values represent significant changes within a group exposed to a differently sexed intruder. D) Linear correlation of large fish bites and intruder bites from the GLMM model with error range in gray. Solid shapes represent same-sex interactions and open shapes represent intersex interactions. E) Reaction norms for intruders in response to the pairing type they were intruding upon based on Number of Bites the intruder had toward the larger fish. For B-E these values represent estimates from the model after all fixed and random effects were considered. Displayed p-values represent significant changes within a group exposed to a different pair type.

## Description

In bi-parental species, task partitioning is an adaptive solution in which parental roles can be divided by sex (Benowitz and Moore 2016; Westrick et al. 2022; McDonald et al. 2024). When individuals are placed in contexts outside the typical arrangement some sex-biased behaviors may show plasticity, sometimes to the point of exhibiting behaviors commonly associated with the other sex, a situation called cross-sexual transfer (West-Eberhard 2003; Anderson and Falk 2023; Anderson and Renn 2023). In some cichlid fishes, biparental units often form pairs with one sex typically larger than the other and distinct roles for each sex (Townshend and Wootton 1985; Snob 2000; Schaedelin et al. 2015). When these fishes lose a partner or are put in a pair that is atypical in its size relationship, the roles shift based on size rather than sex. These shifts can be complete, such that behaviors fully swap between sexes based on size (Awata, Takeuchi, et al. 2006; Wood et al. 2014; Anderson et al. 2025), or partial, such that one or both sexes engage in limited behaviors typical of the other sex (Itzkowitz 1984; Itzkowitz et al. 2005; Snekser and Itzkowitz 2014).

Sex-biased plasticity in territory defense behavior was demonstrated for *Julidochromis marlieri* , a biparental cichlid, for the typical female-larger and experimentally reversed male-larger pairs (Wood et al. 2014). This finding is in line with many other studies within the genus which show that territorial behavior is exhibited by the larger fish (Taborsky and Limberger 1981; Yamagishi and Kohda 1996; Awata and Kohda 2004), yet an unanswered question is the role that the sex of conspecific intruders plays in territory defense. While a heterospecific intruder may represent a similar threat to the reproductive success of both individuals in a pair, a conspecific intruder of the same or different sex represents unique kinds of reproductive threats based on the sex of each member in the pair (Cain et al. 2011; Robart and Sinervo 2018; Trumbo 2022). Male and female *J. marleiri* may likewise have different responses to conspecific intruders. Here, we aimed to address how plasticity of sex-biased behavior interacts with the sex of a conspecific intruder.

We tested the plastic sex-biased behaviors associated with aggression in *J. marlieri* by presenting different sex-larger pairs with sequential intruder challenges of different sexes. It has been suggested that the behaviors of the larger individual in the pair maintain the role of the smaller individual in cichlids (Awata and Kohda 2004; Barbasch et al. 2020), thus a typically paired smaller male *J. marlieri* may not be able to respond to other males should his larger female partner start mating polyandrously (Yamagishi and Kohda 1996). By creating male-larger pairs, we can observe how males engage with conspecific intruders without female interference. We predicted that the larger fish will have greater aggression to intrasex intruders due to the threat they pose to the pair-bond.

We confirmed patterns of previous studies (Awata and Kohda 2004; Awata, Takeuchi, et al. 2006; Wood et al. 2014) in the genus that the larger individual in the pair performed behaviors directed towards the intruder more frequently than their smaller partner, regardless of sex, with biting (GLMM z = -1.969; p = 0.049) as significant, while close-to-divider (GLMM z = -1.690; p = 0.0911) was not. While the power of these findings was low (biting β = 54.2%; close-to-divider β = 43.3%), size differences in behaviors have been confirmed in this species (Wood et al. 2014; Anderson et al. 2025). In all behavioral measures, individuals increased their behaviors for subsequent trials compared to the initial trial, though there were no differences in later trials. An interaction of sex of the larger individual and the sex of the intruder was significant for both behaviors (bite: GLMM z = 2.881; p = 0.004 and close-to-divider: GLMM z = 14.82; p < 0.001) with both having power values over 85%. Males showed a plastic response based on the sex of the intruder by engaging in more aggressive acts towards other males (Fig 1, B-C) while females were consistent in their behavior towards intruders regardless of sex suggesting separate pressures faced by males and females from conspecific intruders (Liker and Szekely 1997; Zimmermann et al. 2021; Trumbo 2022). This imbalance in conspecific defense has been observed in polygynous *Neolamprologus savoryi* where dominant females are more aggressive to male intruders than males are to female intruders, though both aggress more to same-sex intruders (Garvy et al. 2015). In the female-larger species of *Julidochromis,* females are generally more aggressive (Awata and Kohda 2004; Barlow and Lee 2005; Ito et al. 2017), though we found no effect from the sex of the larger fish on its behavior.

As part of courtship, *J. marlieri* are known to bite and seemingly attack potential mates prior to pair bonding (Barlow and Lee 2005). While seemingly an intense aggressive action, biting frequently occurs within established pairs in the genus (Anderson et al. 2025). The inability for researchers to distinguish courtship signals from aggressive signals is a potential confounder in cichlid research (John et al. 2021), as females may be “recruiting” the male intruder by attempting to coerce him into the nest (Awata and Kohda 2004; Barbasch et al. 2020). This ambiguity may explain the apparent lack of adjustment of the larger females’ biting behavior which is supported by the corresponding behavior of the intruder. Our data show that when the intruder experiences more bites from the larger fish they bite more (Fig 1, D), and when that larger fish is the same sex as the intruder the number of bites increases (GLMM t = 2.434; p = 0.024). Since same-sex intruders engage in an equally vigorous bite response this could be a sign of aggression by both individuals. Additionally, female intruders bite more than male counterparts (GLMM t = -2.351 p = 0.029) and while they lessen their attacks on large males, they do not change as drastically as male intruders do (Fig 1, E). This is predicted by female-biased courtship and male-biased choice in polyandrous species (Fritzsche et al. 2021). Even when the relative size-sex relationship is reversed, the direction of these sex-biases is often maintained (Vincent 1994; de Jong et al. 2009). While males play a role in the formation of a pair-bond, active courtship wouldn’t be expected to be part of male’s repertoire if females typically court males (Barlow and Lee 2005). Similar to aggression in convict cichlids (Itzkowitz et al. 2005), *J. marlieri* behavioral roles during pair-bond formation may change in magnitude but not in direction; thus, even as their relative size is reversed, females remain courters and males remain choosers. Our results of female-biased intruder behavior support the hypothesis that while roles in nest defense may undergo cross-sexual transfer (West-Eberhard 2003; Anderson and Falk 2023) in *J. marlieri* , the behaviors related to pair-bond formation maintain their sex-bias.

This finding in *J. marlieri* points to different responses to selective pressures on males and females within a female-larger biparental mating system. Unlike other members of the genus, *J. marlieri* has not been observed to have male-larger pairs (Barlow and Lee 2005) and we were unable to form as many male-larger pairs as female-larger pairs, including the breakdown of one pair prior to the completion of the full trial. Given the consistency of higher female aggression here and other studies (Yamagishi and Kohda 1996; Sunobe 2000; Barlow and Lee 2005), it is challenging to fully assess male response to intruders without female interference. In the female-larger *J. ornatus* , males in polyandrous groups increase testis size (Awata, Heg, et al. 2006), a non-violent form of male-male competition in the face of female aggression. Future work on these fish should consider alternate forms of male-male competition other than aggression, though aggressive acts do appear to play a role in intrasexual conflicts.

## Methods

The Study Population

The *J. marlieri* used in this study were obtained from the hobby trade and maintained in circulating water under conditions to mimic Lake Tanganyika (630-650 µS/cm at pH 8.3, 28 ± 0.3°C) on a 12/12 light/dark cycle with 30 minutes of dusk and dawn and fed flake food to satiation. Tanks included gravel and terracotta tile nests. It is not possible to know how many generations these fish were removed from wild, their exact age, nor the genetic relatedness among individuals, yet breeding captive pairs perform the same types of behaviors as observed in the wild. The advantages afforded by the common garden environment, the ability to control relative size, the ease of observation allowed us to quantify the effect of social environment on pair behavior.

Establishing Pairs

Two mixed sex population tanks (110 L 5-20 fish) were established, one with size bias for larger males and the other with size bias for larger females. When two fish in the community tank displayed pairing behaviors such as nest defense (Wood et al. 2014), they were designated as a pair and were weighed, measured, and had sex confirmed visually (Table 1). Pairs were then moved into the pair compartment of an observation tank (110 L) ( Fig. 1 A) that was divided by an opaque acrylic divider, and further divided by a clear acrylic divider with small perforations for the intruder compartment. Five female larger pairs and three male larger pairs were successfully established and remained paired throughout the experiment. All pairs were acclimated for three to four days. The same individuals remained paired throughout the experiment, though one male-larger pair showed signs of a pair break-down between the third and fourth round. Both individuals displayed stereotypical behavior of an established pair through the third intruder experiment, but we ended this pair early for the safety of the smaller female. Intruders, chosen from a different tank than those used to form pairings, were sexed, measured to always be smaller than the larger fish in the pair, and housed individually.

Behavioral Observation

After the acclimation period, each pair was challenged with an intruder on days 0, 2, 4, and 6. Intruders were introduced five hours after the simulated sunrise with each pair encountering two female and two male intruders in a systematically varied order (Table 1). Video recording with a FujiFilm FinePix S8400W digital camera commenced as the intruder was placed in the small compartment and continued for 10 minutes, after which the intruder was removed. Behaviors were scored using BORIS (Friard and Gamba, 2016) by an observer blind to sex of all fish.

Ethogram

We scored behaviors using an ethogram with three behaviors. “Close-to-divider” was measured as time that each fish spent within one body length of the divider and indicates overall interest in the intruder. “Bite” was scored as the number of times the fish bit or contacted the divider face-first regardless of opponent proximity on the other side of the divider. This same behavior was also scored for the intruders. Given the restricted space and lack of nest this was the only behavior scored for intruders

Statistical Analysis

Data were processed and statistical analysis performed in R version 4.5.1 (R Core Team, 2023) using lme4 (Bates et al. 2015) general linearized mixed effects models (GLMM) with the behaviors of interest as the response variables. Given the evidence that size relative to partner is a significant factor in behavior (Wood et al. 2014), we modeled just the effect of relative size for each behavior to confirm its role. From there we separated out the larger individuals with in the pair from the data set and did independent analysis on the two remaining categorical factors of our fish: sex of the focal individual and sex of the intruder, as well as the interactions of these factors. Since pairs are established and repeatedly exposed to a territorial threat, we also included trial number as an additional fixed effect. Due to the repeated design of the experiment, the focal individual used was included as a random effect. Lastly, since intruders are re-used, we included their identification as a random effect in the model. The behaviors were modeled with a Poisson distribution after assessing the distribution of data. *Post-hoc* pairwise comparisons were run for all four categories.

To analyze the bite behavior of the intruder, we ran a GLMM with bite behavior of both the intruder and larger fish log transformed. We used pair-type (Female-larger or Male-larger) and the intruder’s sex and their interaction as categorical variables. To address social interaction from the perspective of intruder, we included the number of bites by the larger fish in the model since the larger fish engaged in more biting behavior. We used intruder ID and pair ID as the fixed effects. *Post-hoc* pairwise comparisons were run for all four categories. Power analyses were conducted using SIMR (Green and MacLeod 2016) The data used in this study and the code used to analyze them can be found in supplementary files and at the github repository: https://github.com/AndersonDrew/JulidochromisIntruder
